# Shedding Structured Light on Molecular Immunity: The Past, Present and Future of Immune Cell Super Resolution Microscopy

**DOI:** 10.3389/fimmu.2021.754200

**Published:** 2021-12-15

**Authors:** Timothy M. Johanson, Christine R. Keenan, Rhys S. Allan

**Affiliations:** ^1^ The Walter and Eliza Hall Institute of Medical Research, Parkville, VIC, Australia; ^2^ Department of Medical Biology, The University of Melbourne, Parkville, VIC, Australia

**Keywords:** super resolution microscopy, immune cells, immune cell activation, recombination, single molecule microscopy

## Abstract

In the two decades since the invention of laser-based super resolution microscopy this family of technologies has revolutionised the way life is viewed and understood. Its unparalleled resolution, speed, and accessibility makes super resolution imaging particularly useful in examining the highly complex and dynamic immune system. Here we introduce the super resolution technologies and studies that have already fundamentally changed our understanding of a number of central immunological processes and highlight other immunological puzzles only addressable in super resolution.

## Introduction

Microscopy has a long history of enabling immunological discoveries. After likely being observed by the ‘father of microscopy’ A. van Leeuwenhoek in 1687 in human saliva (1), the first definitive description of leukocytes came in 1749 when Joseph Lietaud and Jean-Baptiste de Senac observed human “globuli albicantes” and “globules blanc”, respectively (2, 3). Roughly a hundred years later the first suggestions of immune cell function were observed when leukocytes were seen exiting the vasculature of a frog’s tongue in response to injury (4) and ‘attacking’ a rose thorn stuck into a sea star larva (1).

**Figure 1:**
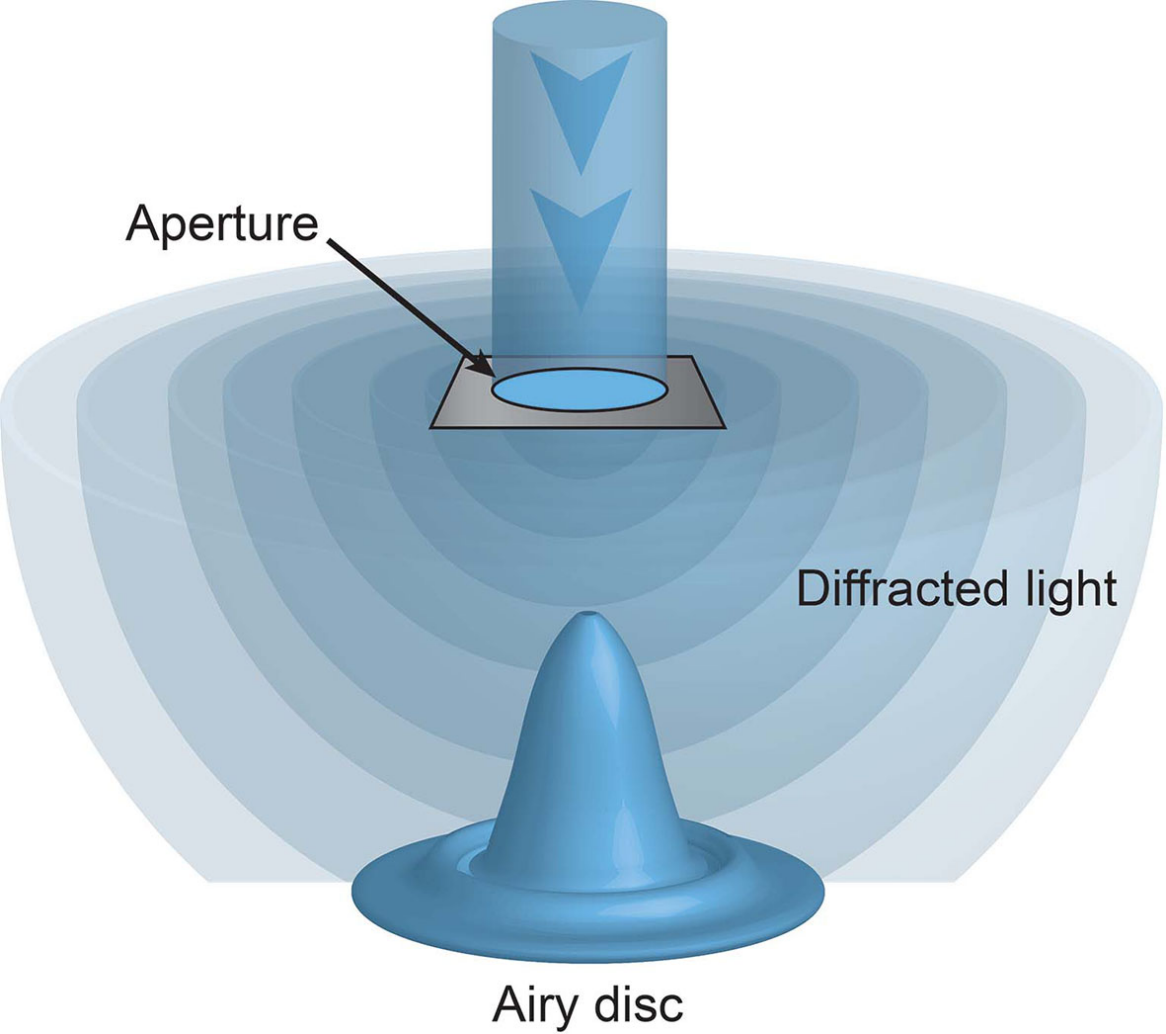
The diffraction limit. Light passing through an aperture diffracts. When hitting a surface this light forms a ripple like pattern of illumination, with a central focus of intensity surrounded by concentric rings, known as an Airy disc. No objects laterally separated by less than the radius of this disc can be discriminated.

Unbeknownst to these microscopy pioneers their ability to observe microscopic structures was limited not only by the strength of light (be it sun or candle) but also the nature of light itself. When light passes through an aperture, such as a microscope objective, it diffracts. How widely it diffracts is dependent on the size of the aperture and the wavelength of the light. Visible light has wavelengths from 400-700 nm. The smaller the aperture or the longer the wavelength of the light the greater the diffraction. When this diffracted light hits a surface, such as the sample, it forms a ripple like pattern, known as an Airy disc (**Figure 1**). The size of the Airy disc is dependent on the extent of diffraction, and importantly sets a limit on the resolution of the microscope. Put simply, only illuminated objects that are laterally separated by more than the radius on the disc, or approximately half the wavelength of the illuminating light, can be discerned. This resolution limit is known as the diffraction limit.

Box 1 **Structural Illumination Microscopy (SIM) (5) uses moveable diffraction gratings inserted into the excitation beam path creating a striped pattern of illumination. By acquiring multiple images with this known pattern of structured illumination it is possible to omit out-of-focus signal to create a super resolution image. SIM can be used to image live cells.**

**Figure:**
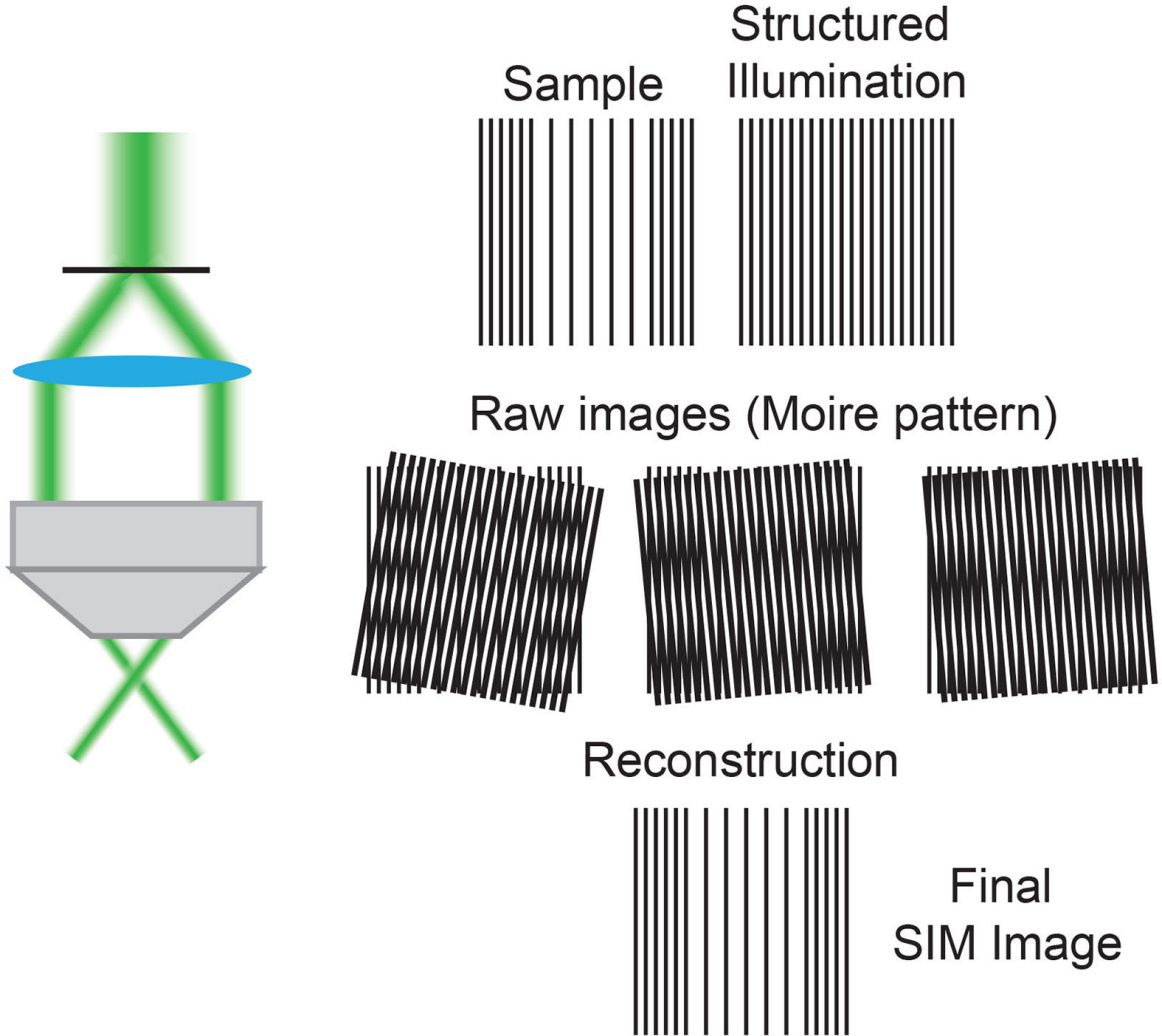

The diffraction limit restricted all forms of light microscopy, including laser microscopy (albeit with a shorter and more defined wavelength), for hundreds of years, until the invention of revolutionary super resolution imaging technologies (5, 6). By structuring the excitation light (**Box 1**) or by using modified laser beams to ‘switch off’ select fluorophores in an illuminated sample (**Box 2**) these pioneering super resolution technologies were able to break the diffraction limit and increase resolution to ~100 nm. Following these breakthrough technologies has a been procession of Nobel Prize winning super resolution imaging technologies that have improved potential resolution to as little as ~20 nm, not only in fixed samples but in highly dynamic live cells and tissues.

The ability to observe and record the behaviour of immune cells, both individually and in tissues, at super resolution has enabled the interrogation of numerous long-standing cellular immunological questions (7). However, while individual cells have been observable for hundreds of years, it was only the super resolution revolution that allowed the thorough examination of single molecules. It is arguably at this molecular level, at which single RNA transcripts (8, 9), individual gene loci (10), chemokines (11), actin filaments (12) and transcription factors (13), among others, can be visually disentangled, that super resolution imaging holds its greatest utility.

Box 2 **Stimulated Emission Depletion (STED) (6) microscopy relies on the interplay between two laser pulses, the first to excite fluorophores at the focal spot, and the second a modified depleting beam that reversibly de-excites any fluorophores surrounding the focal spot. Thus, only the excited fluorophores in the focal spot emit light, allowing features smaller than the diffraction limit to be visualised. STED can be used in live cells.**

**Figure:**
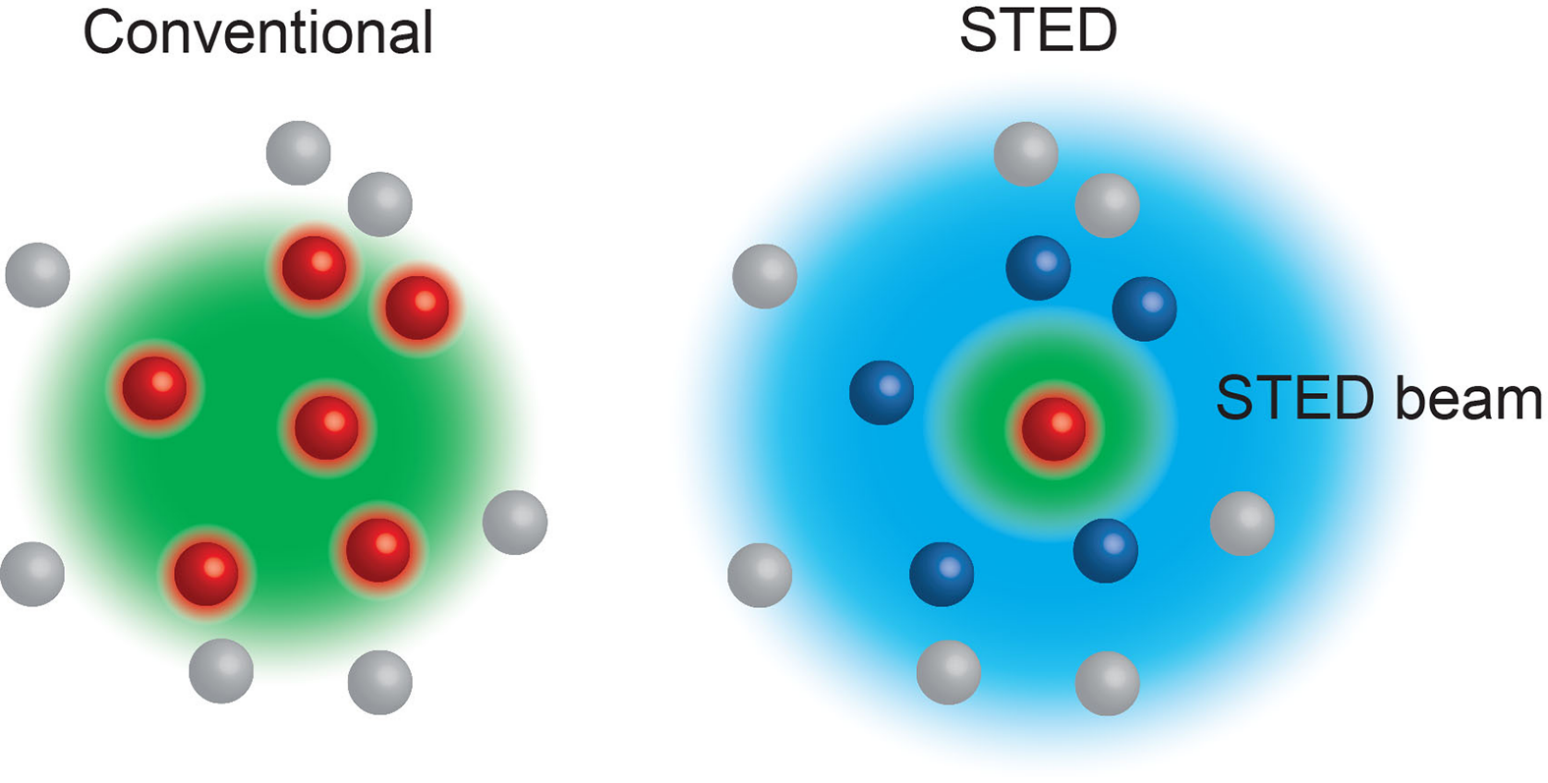

Here we focus on four central immunological processes; two of which super resolution microscopy has already fundamentally changed the way they are understood (immune cell danger detection and activation) and two which these technologies have the currently unrealised potential to answer key, longstanding molecular immunological questions (recombination and lineage decisions). In doing so we also provide introductions to the technologies that have profoundly altered the way not only the immune system, but all life is seen.

### Super Resolution Imaging of Immune Cell Receptors

The ability of immune cells to detect and respond to danger signals is fundamental in immune function. While the receptors involved differ across immune cells types (14) the distribution of these receptors, and other co-stimulatory molecules, is critically important to appropriate activation.

For many years it was thought that receptors, such as B cell receptor (BCR) on B cells, T cell receptor (TCR) on T cells and Toll like receptors (TLRs) on macrophages, were evenly distributed across their respective cell surfaces only to aggregate upon activation (15). However, despite electron microscopy results suggesting the non-random distribution of immunoglobulin molecules on resting B cells (16) as early as 1975, it was not until the advent of super-resolution imaging technologies that it was explicitly shown that many, if not all, receptors cluster within the plasma membrane in the steady state (17–19).

For example, in a seminal work using PALM imaging (**Box 3**) the TCR and a key T cell signalling adaptor molecule, Linker for Activation of T cells (Lat), were shown to reside in clusters upon the plasma membrane, termed protein islands (21). In more recent technically and visually stunning expansions of the characterisation of TCR distribution it was shown that these TCR islands are found across the whole live T cell membrane in culture (22) and in the lymph node (23).

Using other variants of SMLM (**Box 3**) other immune signalling molecules have also been shown to form clusters in the steady state including; CD4 and Lck on T cells (24–26), IgM, IgG and IgD on B cells (27–29), IgE on mast cells (30), TLR4 (31–33), signal regulatory protein α, Fc gamma receptor I and II on human macrophages (34), β2 integrins on human neutrophils (35) and NKG2D on NK cells (36).

Box 3 **Single Molecule Localisation Microscopy (SMLM), including Stochastic Optical Reconstruction Microscopy (STORM) (20) and Photo-Activated Localization Microscopy (PALM) (17), use a low power beam to activate a small proportion of reversibly photoactivatable molecules within an illuminated area before a higher power illuminating beam records the molecules position and photobleaches them. As only a small proportion of the total fluorescent molecules are activated in each cycle the centre of mass of individual molecules can be determined in each image (a process that would be impossible if all molecules fluoresced simultaneously) before being collated into a final super resolution image. These compiled images can achieve ~25 nm resolution. PALM generally use genetically encoded photo-switchable fluorescent proteins, while STORM uses conventional synthetic dyes. Both can be used in live cells.**

**Figure:**
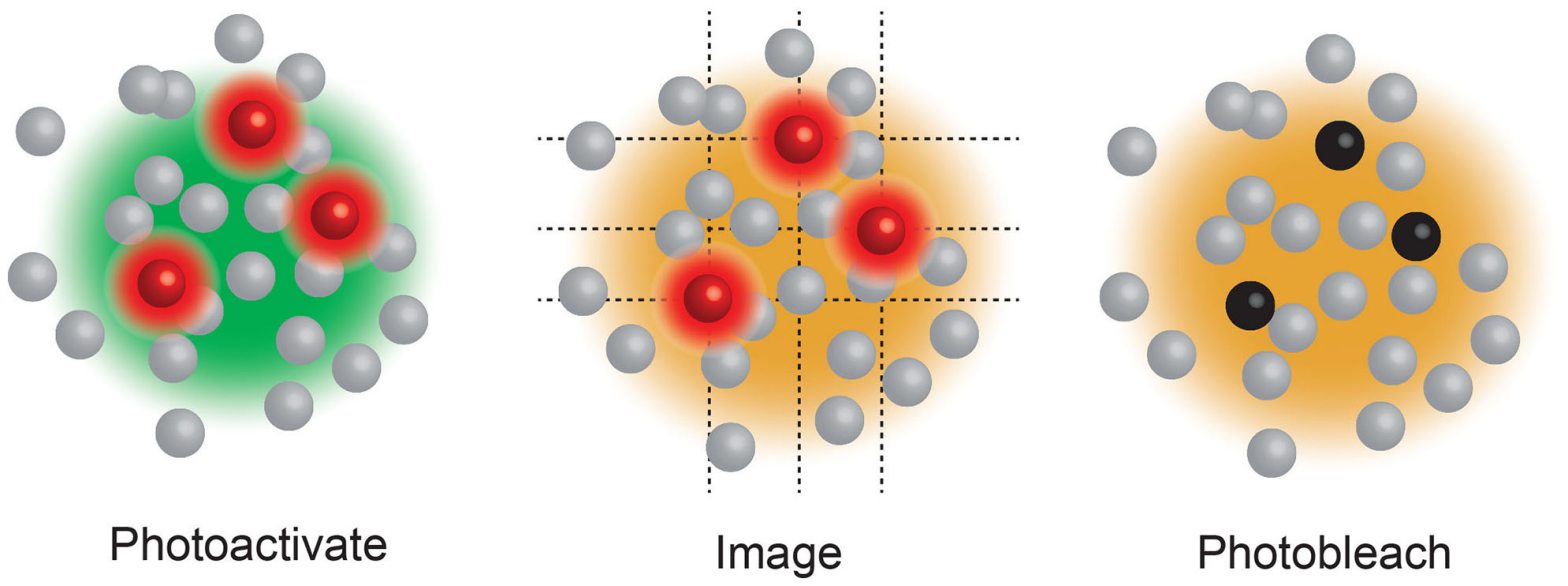

**Figure 2:**
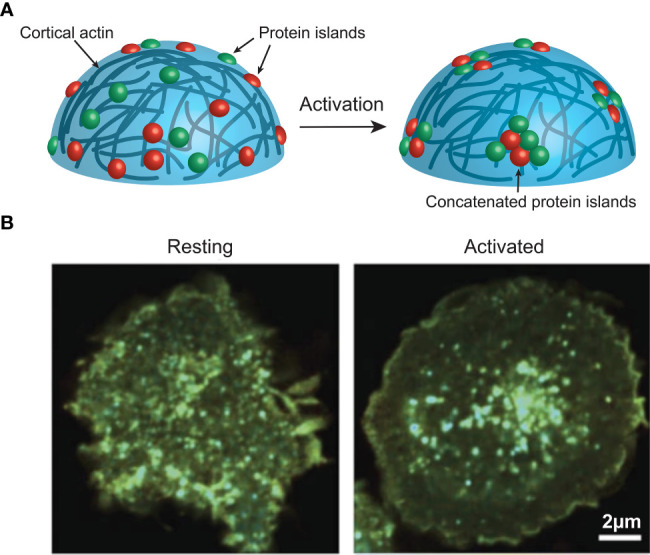
**(A)** Activation induces actin-mediated concatenation of cell surface protein islands on the surface of immune cells to facilitate signalling. **(B)** FRET imaging data from Ma et al. (38), showing CD3z clustering on a Jurkat cell before and after activation. Image used under the terms of the Creative Commons licence.

The majority of these studies revealed not only the pre-activation clustering of these signalling molecules, but a consistent activation induced redistribution of these clusters. Interestingly, evidence from STORM, PALM and FLIM/FRET (**Box 4**) imaging of T and B cell membranes reveals this redistribution to be a concatenation, but not coalescence, of these clusters (21, 38, 39) (**Figure 2**). This concatenation of protein islands, as opposed to a complete merging, is thought to play an important regulatory function. As such, it is thought that signalling occurs only at the boundaries of clusters that contain distinct compositions of important signalling molecules. For example, in B cells IgM and CD45 are found together on an island separated from islands containing Lyn and CD19 (40, 41). This is important as Lyn is required for some forms of signalling *via* IgM (42, 43). Thus, the two islands must come together, and exchange components, during activation, however, if complete coalescence of the islands was allowed dysregulated activation could result.

Box 4 **Fluorescence-lifetime imaging microscopy (FLIM) (37) images the decay rate of fluorescence of a tagged donor molecule of interest after excitation. This rate of decay is impacted by the proximity of an acceptor molecule. The closer the molecules of interest, the faster the decay. As such, FLIM-FRET techniques provide high temporal resolution of tagged protein-protein interactions in live cells.**

**Figure:**
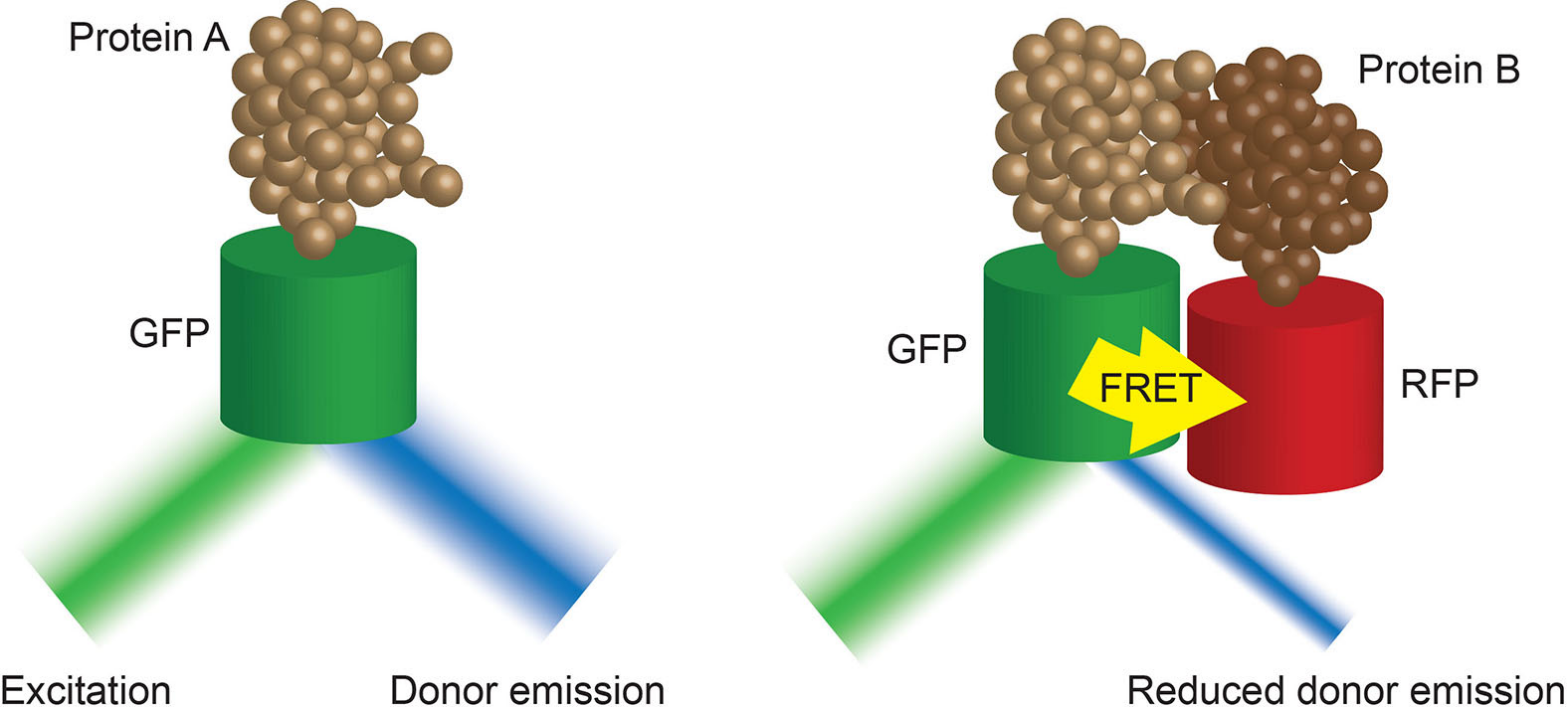

It has been known for over a decade that the network of cortical actin just under the cell membrane plays a critical role in the separation of protein islands (44, 45). However, it was the application of super resolution imaging technologies that allowed elucidation of the underlying molecular mechanism of control. As such, STORM imaging has shown that treatment of B cells with an actin-depolymerizing compound (latrunculin A) increases the proximity of the aforementioned IgM and IgD containing islands (28) and the lateral mobility of BCR and CD19 containing islands (27, 46, 47).

### Immune Cell Activation at Super Resolution

Once a danger signal is detected, immune cells undergo dramatic cellular and molecular changes in order to play their part in the immune response. In addition to revealing previously unseen mechanisms of immune cell danger detection, super resolution imaging has also added to our understanding of the molecular changes during immune cell activation.

Some immune cells, such as cytotoxic T cells and natural killer (NK) cells, respond to activation with the release of lytic granules at a synapse between them and their target cell. These granules are designed to induce apopotosis in the target cell. Unsurprisingly, given their lethality, the formation, trafficking and release of these modified lysosomes is tightly controlled. The ability of sub-diffraction limit imaging to visually untangle the dense, intricate and highly dynamic network of cortical actin and lytic granules underlying the synapse and the plasma membrane in general has revolutionised our understanding of immune cell killing (48).

**Figure 3:**
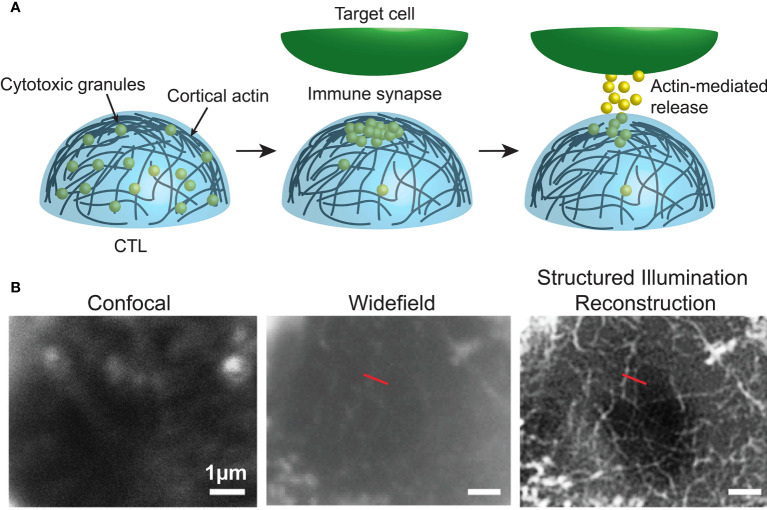
**(A)** Dynamic cortical actin regulates the accumulation and release of cytotoxic granules at the immune synapse of cytotoxic immune cells. **(B)** Data from Brown et al. (57), comparing F-actin (white) at human NK cell synapses using confocal, widefield and structured illumination reconstruction imaging. Image used under the terms of the Creative Commons licence.

For example, in recent years a number of super-resolution imaging technologies, including 3D-SIM (49), STED (50, 51), SMLM and TIRF (**Box 5**) (53, 54), and Lattice light sheet microscopy (**Box 6**) (12, 56) have all been used to observe the rapid and intricate movement of actin and lytic granules towards, and within, the immune synapse of both T and NK cells. As such, it was revealed that upon activation the network of actin that normally forms a mesh too dense for lytic granules to traverse dilates or dissolves at the immune synapse allowing microtubule-guided granule release (**Figure 3**) This process takes approximately one or thirty minutes in T and NK cells, respectively (50, 54, 57).

Box 5 **Total internal reflection fluorescence microscopy (TIRF). When light encounters the interface of two transparent materials with different refractive indices (such as a live cell and a cover slip, as below), it will most often be both diffracted and reflected. However, at a certain angle of incidence the light will be totally reflected in a phenomenon called total internal reflection. Total internal reflection creates an electromagnetic field that passes through the interface between the two materials to form an evanescent wave. TIRF imaging (52) exploits this evanescence to excite fluorophores only in close proximity to the interface to achieve sub-diffraction limit axial resolution.**

**Figure:**
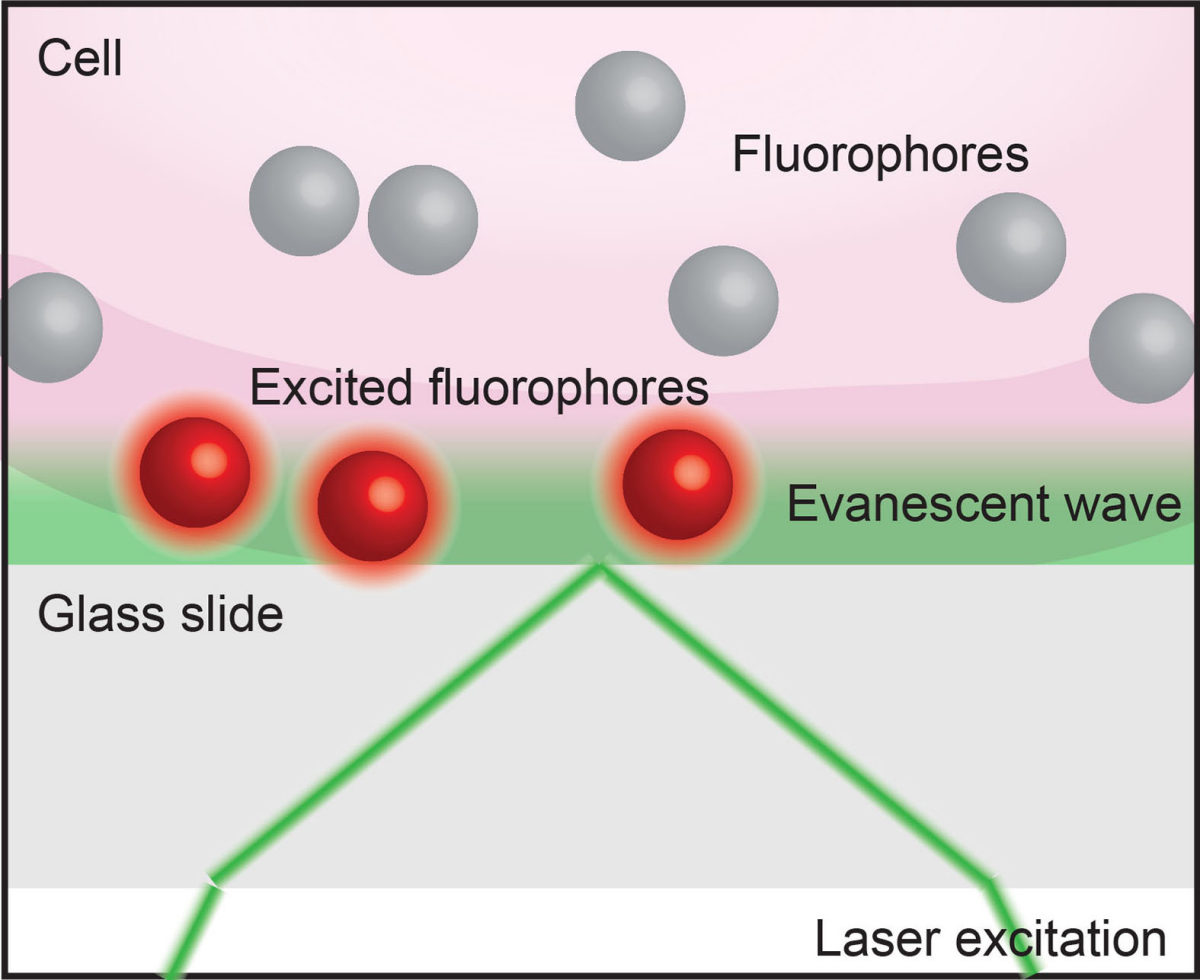

Furthermore, not only is the intricate actin network important for facilitating degranulation, it also appears to play an important role in regulating the number of granules released. This control is critically important as it minimises healthy by-stander cell killing (58) and potentially also influences the number of target cells an individual cytotoxic lymphocyte can kill (59). Recent imaging studies have shown that T and NK cells do not release their entire granule payload during initial degranulation. In fact, they may release as little as one tenth of their total granules (60, 61). Actin likely regulates degranulation *via* two mechanisms; one, it limits the transport of granules to the immune synapse (60) and two, TIRF imaging recently observed the reformation of the dense actin network underlying the immune synapse soon after degranulation, restricting further granule release (12).

While cytotoxic lymphocytes respond to activation with the direct killing of target cells, B lymphocytes direct killing *via* the mass production of specific antibodies. This requires transformation into ‘antibody factories’, including dramatic increases in cell size, proliferation and RNA synthesis (62). The process also involves the spreading of chromatin from its naïve location, predominantly at the nuclear periphery, to a more dispersed configuration. This chromatin spreading is thought to promote transcription factor binding and gene expression important in the transformation to antibody secreting cells (63, 64).

Box 6 **Lattice light sheet fluorescence microscopy (55) uses a combination of techniques from light sheet, Bessel beam and structural illumination microscopy (SIM). As such, it uses a two-dimensional lattice of non-diffracting Bessel beam light sheets that are spaced such that they cause destructive interference and removal of the ‘out of field’ illumination which hampers traditional Bessel beam light sheet microscopy. This allows lattice light sheet microscopy to achieve unparalleled resolution and penetrance, while minimising phototoxicity.**

**Figure:**
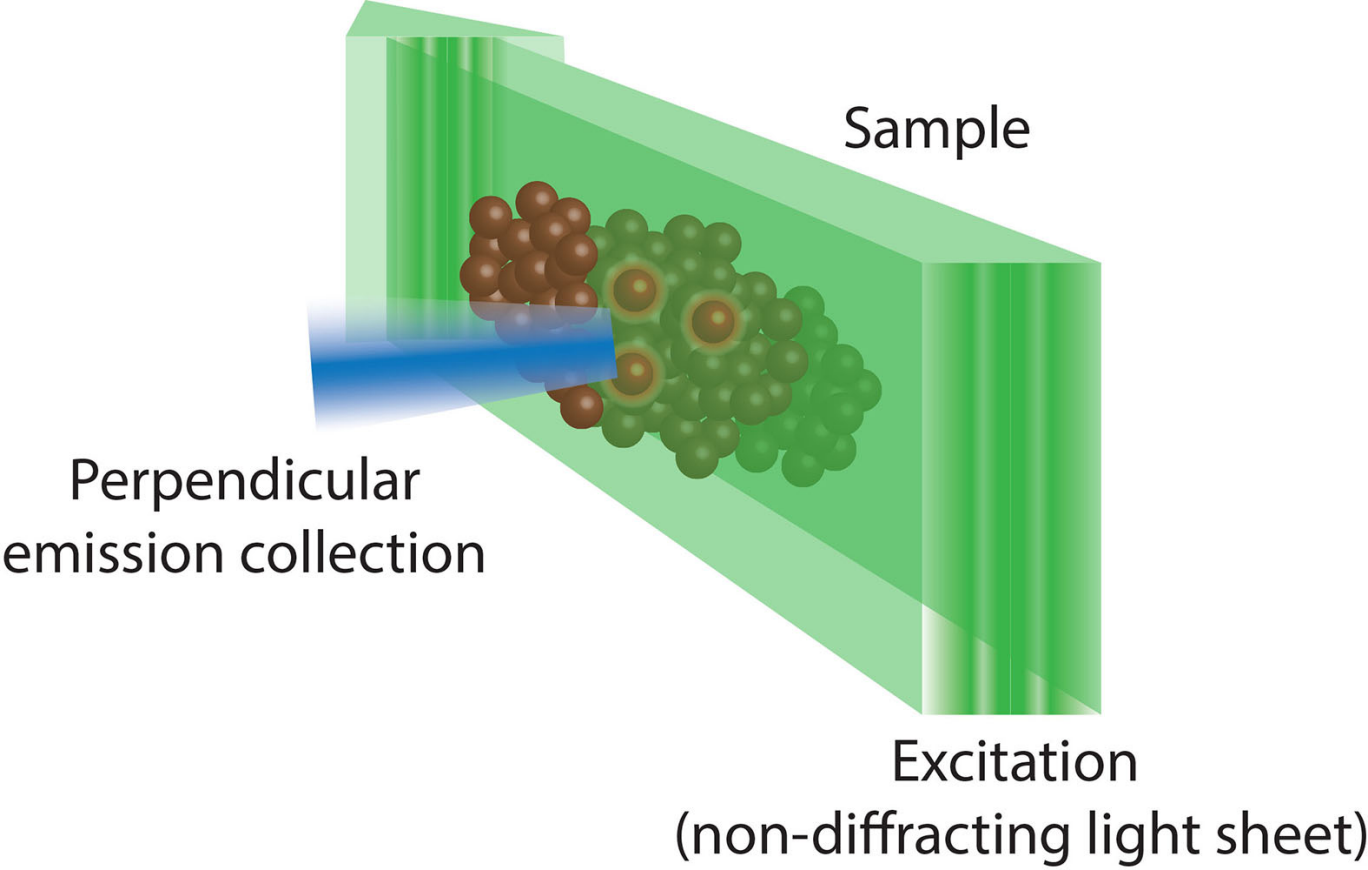

While super resolution investigations confirmed chromatin spreading (13, 65), the ability to visualise the chromatin fibre to <20 nm resolution revealed that not only do the fibres spread within the nucleus, they also decompact, meaning there is more accessible DNA between normally tightly compacted nucleosomes. These processes were shown to be regulated independently, and furthermore it was chromatin decompaction, not spreading, that was important in regulating transcription factor binding (13). By inserting a fluorescent Halo-Tag downstream of two transcription factors, CTCF and JunD, almost unimaginably detailed three-dimensional single molecule tracking revealed the binding and diffusion behaviours of these factors during their DNA interrogations. As such, in a naïve B cell JunD collides with DNA roughly 130 times before finding a suitable and accessible binding site. This search time is roughly halved upon B cell activation (13). This was elegantly shown to be independent of chromatin spreading and reliant upon nucleosome decompaction using drug treatment or energy depletion, respectively (13). The dwell or residence times of CTCF determined by single molecule tracking was confirmed using FRAP imaging (**Box 7**).

Box 7 **Fluorescence recovery after photobleaching (FRAP) (66) measures the recovery of local photodestruction of a tagged molecule via diffusion to determine the molecules dynamics within the local molecular environment.**

**Figure:**
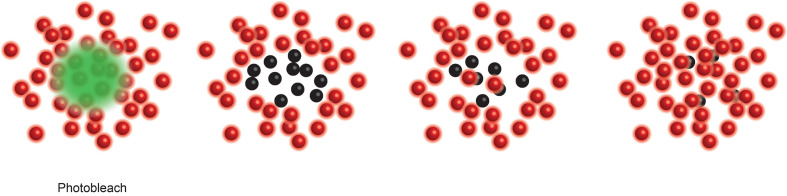

These studies are examples of how super resolution imaging has already fundamentally changed our understanding of central immunological processes, in this case by revealing the molecular underpinnings of immune cell activation. While earlier technologies did elude to many of these mechanisms, the fact that actin fibres, nucleosomes and indeed protein islands (22) are frequently separated by less that 200 nm means they can only be meaningfully visualised, and thus more completely understood, in super resolution.

## The Future of Super Resolution in Molecular Immunology

The works outlined thus far highlight the impact of super resolution imaging on our understanding of immune cell function. While these explorations have already yielded fruit, below we outline two incompletely understood, but essential, molecular immune processes in which super resolution imaging has the potential to answer longstanding questions.

### Antigen Receptor Recombination

Recombination of the antigen receptor genes (*Igh, Igk and Igl* in B cells and *Tcrg*, *Tcrd*, *Tcrb* and *Tcra* in T cells) is key to generating a wide antigen receptor repertoire. The tightly controlled process involves removal of the intervening DNA between genes from three segment pools, known as variable (V), diversity (D) and joining (J). The result is in an exon that encodes the antigen-binding domain of an antigen receptor. Recombination relies on a series of remarkable genomic manoeuvres, including relocalisation of the antigen receptor gene from the periphery to the centre of the nucleus (67, 68), removal of genomic domain boundaries within the gene (69) and a contraction of the gene to bring linearly distant V regions into close physical proximity with the D-J region for recombination (68, 70–72). These processes have been extensively examined using molecular and genetic manipulations, but also imaging technologies. For example, DNA FISH has been used to quantify the nuclear position and contraction of the Igh locus in B cell progenitors (67).

While these studies have added to our understanding of antigen receptor recombination there are still significant gaps in our knowledge of the process. For example, while locus contraction brings the V region into proximity with the D-J region, how the V region that will ultimately form part of the functional exon is ‘selected’ from numerous candidates within the distal region is unclear. Diffusion fitting a fractional Langevin motion model (73) within the viscoelastic nuclear environment is currently the best explanation of how this process may work (74, 75).

Recent super resolution imaging breakthrough technologies provide an opportunity to reveal the mechanics of this long-standing immunological puzzle. These technologies, including ORCA and Hi-M (**Box 8**) (80, 81), leverage the development of complex pools of synthetic fluorescently labelled oligonucleotides (OligoPaint) alongside sequential super resolution STORM imaging to reveal the nanoscale configuration of genomic regions. In visually stellar works building on super resolution examinations of chromosome scale genome organisation (83–85), both ORCA and Hi-M have been used to visualise the nanoscale (2-15 kB resolution) organisation of specific genomic regions (up to 700 kB in size) within individual cells of whole *Drosophila* embryo sections. These works revealed previously undetectable relationships between genome organisation, epigenetic states and transcription (80, 81).

Box 8 **While visualisation of total RNA or DNA is relatively trivial, identification of specific RNA species or DNA regions within the vast cellular pool of both is far from it (76). The visualisation of RNA is most frequently used to identify transcriptional activity based upon concentrations of specific RNA species. For many years, RNA Fluorescence *In Situ* Hybridization (FISH) (77) was the method of choice. However, the number of different RNA species detected was limited by fluorescence spectra. Recent multiplexing technologies resolved this issue using sequential imaging allowing visualisation of over a thousand RNA species in a single cell (8, 9). While FISH requires sample fixation, there are a number of imaging technologies that allow visualisation of RNA in live cells. These utilise small molecule fluorescent dyes (molecular beacons, nanoflares and dye aptamers) or fluorescent proteins fused to RNA aptamer binding proteins (MS2, PP7 or pumilio1) or single stranded RNA-binding Cas9 (rCas9) (78). The aptamer strategies require genetic engineering of the RNA of interest to insert aptamer sequences while beacons, nanoflares and rCas9 bind native RNA species. Similar to RNA visualisation, DNA FISH (79) has traditionally been the method used to view locus position within fixed cells, with the same spectral constraints. Recent technological advances have allowed both sequential imaging in fixed cells [ORCA (80), Hi-M (81)] and imaging DNA in live cells (10, 82). Similar to the multiplexed RNA-FISH technologies the DNA sequential imaging technologies use successive rounds of imaging separated by fluorescent stranddisplacement to reveal the location of, and relationship between, numerous regions of DNA. Imaging DNA regions of interest in live cells currently relies upon the binding of tagged and catalytically dead Cas9 (dCas9) to these loci. This creates challenges in delivering sufficient guide RNAs to target labelled dCas9 to the regions of interest. One recent solution includes the development of molecular assembly strategies that allow the introduction of up to 36 guide RNAs into a single cell providing sufficient guide to visualise non-repetitive DNA regions in live cells (10).**

**Figure:**
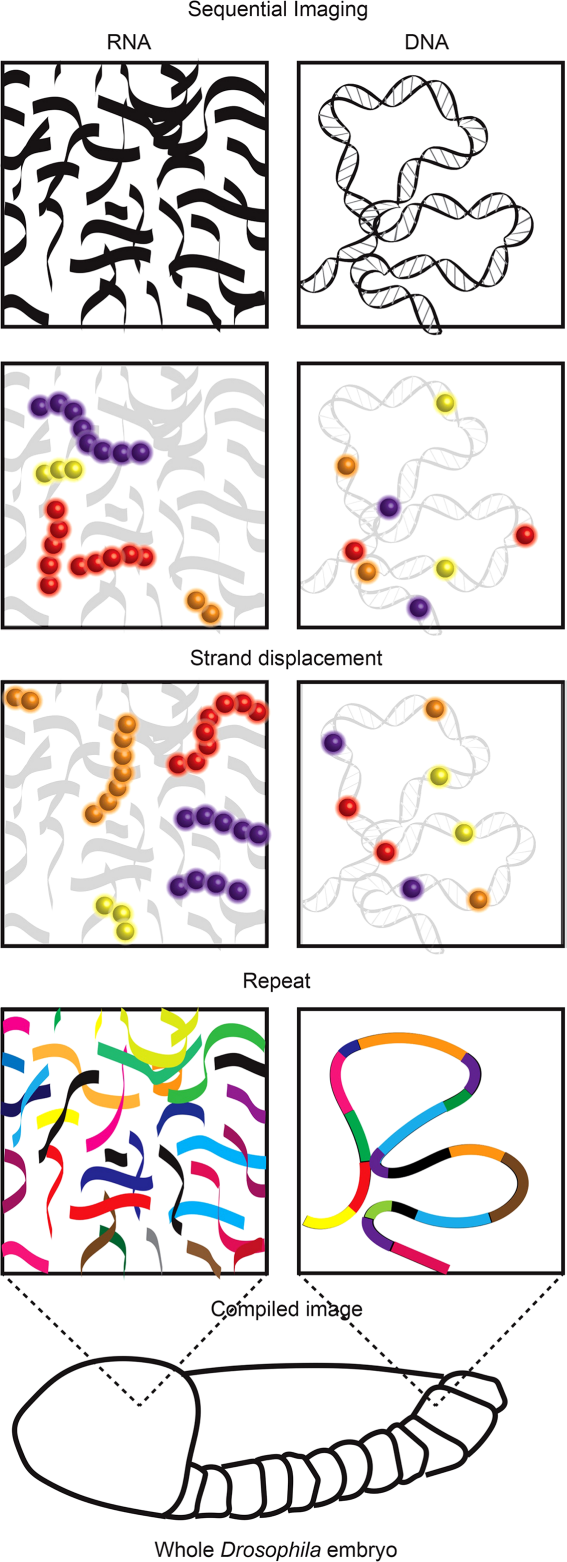

Given the unprecedented resolution, throughput and applicability of these new super resolution technologies it is conceivable that they could be used to examine the nanoscale genome organisation of antigen receptor loci in thousands of adaptive immune cell progenitors of any species. Taking mouse *Igh* as an example 700 probes would be sufficient to cover the entirety of the expansive 2 million base pair locus at a 3 kb resolution. Given the 113 V_H_ region gene segments are mostly separated from each other by at least 5 kb (86), 3 kb resolution would be sufficient to reveal the location of all V_H_ gene segments, along with the rest of the locus, within thousands of individual cells. While fixation required by sequential imaging prohibits a live view of the recombination process, a compilation analysis from the thousands of single cells could reveal an unparalleled view of the local genomic environment in which recombination occurs (80, 84, 87). This could reveal patterns of order, indicative of stable, consistent position or interactions, or disorder, potentially revealing regions undergoing random diffusion. For example, it could be that locus contraction consistently brings particular regions into physical proximity, relative to all others. Alternatively, it could be that the entire locus diffuses with minimal physical constraints and no discernible patterns of interaction. Either way these breakthrough super resolution technologies could enable a greater understanding of the role of diffusion and physical proximity during recombination.

As for live imaging, the fixation required for ORCA and Hi-M would also obstruct downstream examination of the physiological impacts of the visualised genomic organisation. For example, even if as hypothesised the distal V_H_ gene segments are revealed to contract to, then diffuse near, the D-J region, the ultimately selected segment could not be confirmed within fixed cells. However, here it is worth remembering that the near universal applicability of these technologies mean they can not only expand our understanding of steady state conditions, but also be applied to the genetic and molecular manipulation systems used previously to understand recombination. Thus, perturbations to antigen receptor loci genomic organisation could be re-examined using these novel technologies validating and expanding previous conclusions.

### Immune Cell Lineage Decisions

Immune cells make up arguably the most diverse cellular system in complex organisms. This diversity requires numerous lineage decisions as an immune cell differentiates from a haematopoietic stem cell. Be they step-wise and absolute or fluid and continuous (88), these decisions are directed by transcription factors (89). Some of these factors as so influential that the expression of a single transcription factor gene can alter a cells lineage fate (90, 91). The cellular consequences of these lineage decisions have been well explored, in part using imaging (7). However, the molecular events underlying immune cell lineage decisions remain largely unexplored.

Recent application of cutting-edge super resolution imaging technologies in other systems have demonstrated the power of these technologies to reveal molecular insights into transcriptional regulation, and thus potentially lineage decisions. For example, in a recent technical masterpiece single molecule tracking combined with target loci locking microscopy was used to reveal the single molecule resolution, real time kinetics of transcription and its regulators in mouse embryonic stem cells (92). As such, phage genome sequences that can be recognised by fluorescently tagged phage coat proteins were engineered into the 3’ UTR of two pluripotency transcription factor genes (**Box 8**). This allowed single molecule visualisation of nascent mRNA. In the same cells, RNA polymerase II or other transcriptional regulatory factors (Sox2, Cdk9, Brd4 or Mediator) were fluorescently labelled. This allowed a phenomenally detailed examination of the relationship between the numbers, dynamics and positioning of these factors relative to transcription, revealing hierarchical, highly dynamic (2-10 second turnover) but relatively small clusters (<20 molecules) of all factors at sites of transcription.

As mentioned above single molecule tracking has been previously performed in immune cells (13). However, these experiments were not in the context of lineage decisions or concurrent with transcriptional visualisation. Here we outline experiments applying the visualisation systems used in *Drosophila* transcriptional regulation to immune cell lineage decisions. While ultimately these experiments could be conducted in genetically engineered primary cells, there are numerous *in vitro* systems in which immune cells can be induced to make lineage decisions. For example, the monocytic cell line THP-1 can be induced to differentiate into M1 or M2 macrophages by treatment with propidium monoazide (93). Within this system expression of lineage defining transcription factors, such as STAT1, 3 or 6, could be visualised (94). When the expression of these transcription factors is first detected the locus could be target locked and the relationship between transcription and single molecules of select regulatory factors could be examined. This could reveal how single molecules can regulate expression of these lineage defining transcription factors, and thus influence the fate of the immune lineage.

One obvious weakness of this methodology is the inability to visualise regulatory events prior to transcription initiation. Many of these events are likely just as lineage defining as those after transcription begins. As previously outlined, there are a number of technologies that allow visualisation of specific loci in cells (**Box 8**), however, none have yet been combined with live single molecule tracking of regulatory factors and transcription. Like so many recent molecular technologies one recent breakthrough in visualising loci in live cells utilises catalytically dead Cas9 (dCas9). As such, in a system known as Chimeric Array of gRNA Oligonucleotides (CARGO), numerous guide RNAs are introduced into the cell to guide fluorescently tagged dCas9 to a locus of interest (10) (**Box 8**). While the presence of dCas9 was shown not to dramatically impact local genome organisation (10), it is likely that dCas9 will obstruct other regulatory factors at sites of interest. Thus, other methods of visualising loci of interest prior to transcription will be required if the regulatory events prior to transcription are to be studied at the nanoscale.

While there is still work to be done, recent developments in super resolution imaging have revealed the behaviours of lineage defining molecules, be it transcriptional regulators or genomic loci, in almost unimaginable detail. If, or perhaps when, they are ultimately applied to immune cells, these single molecule scale technologies will provide an unprecedented view of entire antigen receptor gene loci and potentially allow us to watch as a single transcriptional regulator changes the fate of an entire lineage.

## Conclusion

In the two decades since the invention of laser-based super resolution imaging, scientists have used these technologies to continue the long tradition of using microscopy to understand the immune system.

However, while impactful, all of these discoveries have been made using *in vitro* systems. This is because *in vivo* super resolution technologies still face major technological hurdles. The solution will likely come by emulating current high-resolution *in vivo* imaging systems. These high-resolution systems, such as confocal microscopy, have used surgically implanted windows (95, 96) or simply exteriorized, though still living, organs and tissues in reveal important insights into immune cells *in vivo*. Among many insights, high-resolution imaging has revealed distinct waves of cancer-induced immune cell infiltrates (97) and the role of neutrophils (98, 99), macrophages (100) and dendritic cells (101) in combatting, but also at times inadvertently aiding, cancer progression. Furthermore, the speed of high-resolution imaging technologies has allowed the imaging of interactions between immune cell types in real time. For example, using intravital microscopy in exteriorized lymph nodes of anesthetized mice, Mempel et al. tracked how cytotoxic T cells interacted with antigen-presenting B cells in the presence or absence of regulatory T cells in real time (102). Other examples include interactions between NK cells and dendritic cells (103), macrophages and dendritic cells (104), macrophages and cytotoxic T cells (105), among many others (106).

Finally, and perhaps most clinically relevant, high-resolution *in vivo* imaging has allowed tracking of the immune cell response to drug treatment. For example, Hawkins et al. imaged the retraction of T cell leukaemia in the calvarium of the mouse skull upon dexamethasone treatment (95), while Lohela et al. imaged the reduction in macrophages and dendritic cells in the mouse mammary gland during anti-colony stimulating factor 1 treatment (107).

Expanding these types of studies to super-resolution imaging has significant further challenges. These include scattering of structured light by dynamic tissues (108) to balancing excitation power to detect nanoscale structures while avoiding lethal phototoxicity. However, new technologies continue to push these boundaries (80, 81), often by combining the strengths of existing systems, such as lattice light sheet microscopy and adaptive optics (109). Currently the financial and technical thresholds of these technologies mean they are not widely available; however, excitingly, it is likely that immunologists will soon be able to use these and other, as yet unimagined, technologies to explore nanoscale structures within living tissues. Thus, the future of super resolution imaging is bright and will continue to shed (structured) light on molecular immunology well into the future.

## Author Contributions

TJ, CK and RA wrote the manuscript. All authors contributed to the article and approved the submitted version.

## Funding

This work was supported by grants and fellowships from the National Health and Medical Research Council of Australia (TJ #1124081, RA and TJ #1049307, #1100451, CK #1125436). This study was made possible through Victorian State Government Operational Infrastructure Support and Australian Government NHMRC Independent Research Institute Infrastructure Support scheme. The funders had no role in the decision to publish or preparation of the manuscript.

## Conflict of Interest

The authors declare that the research was conducted in the absence of any commercial or financial relationships that could be construed as a potential conflict of interest.

## Publisher’s Note

All claims expressed in this article are solely those of the authors and do not necessarily represent those of their affiliated organizations, or those of the publisher, the editors and the reviewers. Any product that may be evaluated in this article, or claim that may be made by its manufacturer, is not guaranteed or endorsed by the publisher.
